# Antenatal Dexamethasone after Asphyxia Increases Neural Injury in Preterm Fetal Sheep

**DOI:** 10.1371/journal.pone.0077480

**Published:** 2013-10-18

**Authors:** Miriam E. Koome, Joanne O. Davidson, Paul P. Drury, Sam Mathai, Lindsea C. Booth, Alistair Jan Gunn, Laura Bennet

**Affiliations:** Department of Physiology, the University of Auckland, Auckland New Zealand; Fudan University, China

## Abstract

**Background and Purpose:**

Maternal glucocorticoid treatment for threatened premature delivery dramatically improves neonatal survival and short-term morbidity; however, its effects on neurodevelopmental outcome are variable. We investigated the effect of maternal glucocorticoid exposure after acute asphyxia on injury in the preterm brain.

**Methods:**

Chronically instrumented singleton fetal sheep at 0.7 of gestation received asphyxia induced by complete umbilical cord occlusion for 25 minutes. 15 minutes after release of occlusion, ewes received a 3 ml i.m. injection of either dexamethasone (12 mg, n = 10) or saline (n = 10). Sheep were killed after 7 days recovery; survival of neurons in the hippocampus and basal ganglia, and oligodendrocytes in periventricular white matter were assessed using an unbiased stereological approach.

**Results:**

Maternal dexamethasone after asphyxia was associated with more severe loss of neurons in the hippocampus (CA3 regions, 290±76 *vs* 484±98 neurons/mm^2^, mean±SEM, P<0.05) and basal ganglia (putamen, 538±112 *vs* 814±34 neurons/mm^2^, P<0.05) compared to asphyxia-saline, and with greater loss of both total (913±77 *vs* 1201±75/mm^2^, P<0.05) and immature/mature myelinating oligodendrocytes in periventricular white matter (66±8 *vs* 114±12/mm^2^, P<0.05, *vs* sham controls 165±10/mm^2^, P<0.001). This was associated with transient hyperglycemia (peak 3.5±0.2 vs. 1.4±0.2 mmol/L at 6 h, P<0.05) and reduced suppression of EEG power in the first 24 h after occlusion (maximum −1.5±1.2 dB vs. −5.0±1.4 dB in saline controls, P<0.01), but later onset and fewer overt seizures.

**Conclusions:**

In preterm fetal sheep, exposure to maternal dexamethasone during recovery from asphyxia exacerbated brain damage.

## Introduction

Fetuses at risk of premature delivery are now routinely exposed to maternal treatment with synthetic glucocorticoids. Preterm infants have a high rate of neuronal and white matter damage [1], [2]. Clinically, maternal glucocorticoids substantially reduce acute neonatal morbidity and mortality and reduce intraventricular hemorrhage [3]. However, although they reduce the risk of white matter injury [4], [5], the effect on later neurodevelopmental outcome is still unclear [6], and some studies report reduced head size [7].

Some of the apparent inconsistency may be related to the effect of glucocorticoids on the brain after exposure to hypoxia-ischemia (HI). For example, in preterm infants dying within 4 days after birth, exposure to maternal steroid therapy was associated with reduced hippocampal neuronal density [8]. Critically, there is now compelling evidence that early loss of vulnerable cells such as pre-oligodendrocytes is followed by chronic impairment of white and grey matter maturation [2], [9], [10]. Thus it is vital to understand how maternal glucocorticoids affect recovery from perinatal brain injury. Studies in newborn rodents suggest little effect of glucocorticoids after HI, as reviewed [11]. In near-term fetal sheep, at an age when brain maturity is broadly equivalent to term infants, maternal dexamethasone treatment 48 hours *before* cerebral ischemia did not modify the pattern of injury [12]. However, perhaps surprisingly, there is no information on how glucocorticoids after preterm HI affect the pervasive white and grey matter injury that underpins long-term impairment of neurodevelopment [1], [2].

We recently reported that in normoxic preterm fetal sheep a standard clinical course of maternal dexamethasone was associated with significant transient EEG hyperactivity from approximately 3 hours after the first injection [13]. Although there was no cerebral injury after 5 days of recovery, loss of neural suppression after hypoxia-ischemia can be associated with increased neural injury [14], [15].

Therefore, we examined the hypothesis that maternal dexamethasone therapy shortly after a period of severe asphyxia would increase fetal neural activity during the early recovery (‘latent’) phase, and increase neural injury. These studies were conducted in preterm fetal sheep at an age that is broadly equivalent in brain maturation to the 27–30 week human [16].

## Materials and Methods

### Animals and experimental procedures

All procedures were approved by the Animal Ethics Committee of the University of Auckland. Singleton Romney/Suffolk fetal sheep were surgically instrumented at 98–100 days of gestation (term = 147 days). Ewes were anesthetized by intravenous injection of propofol (5 mg/kg, AstraZeneca Limited, Auckland, New Zealand), followed by 2–3% isofluorane in oxygen. A midline incision was made to expose the uterus, and the fetus was partially exteriorized for instrumentation. Polyvinyl catheters were placed in the amniotic sac, left femoral artery and vein and right brachial artery to measure blood pressure and for pre-ductal blood sampling. Two pairs of electrodes (Cooner Wire, Chatsworth, CA, USA) were placed over the parietal cortex bilaterally, 10 mm lateral to bregma and 5 mm and 10 mm anterior to measure electroencephalographic (EEG) activity. A reference electrode was sewn over the occiput. A pair of electrodes was placed across the fetal chest to measure the fetal electrocardiogram (ECG). In addition, an inflatable silicone occluder was placed around the umbilical cord (In Vivo Metric, Healdsburg, CA, USA). All fetal leads were exteriorised through the maternal flank, and a maternal leg vein was catheterised for post-operative care and euthanasia.

Antibiotics were administered into the amniotic sac (80 mg Gentamicin, Pharmacia & Upjohn, Rydalmere, NSW, Australia) before the uterus was closed. Ewes were given 5 ml of Streptocin (Stockguard Labs Ltd., Hamilton, New Zealand) i.m. 30 min before surgery for prophylaxis. The maternal midline skin incision was infiltrated with a local analgesic, 10 ml 0.5% bupivacaine plus adrenaline (AstraZeneca Ltd., Auckland, New Zealand). After surgery, ewes were housed together in separate metabolic cages with *ad libitum* access to food and water. Rooms were temperature and humidity controlled (16 ± 1°C, humidity 50 ± 10%) with a 12 h light/dark cycle (light 0600 to 1800 h). Ewes were given daily i.v. antibiotics (600 mg Crystapen, Biochemie, Vienna, Austria and 80 mg Gentamicin) for 4 days after surgery. Fetal catheters were maintained patent with continuous infusion of heparinised saline (20 U/ml). Experiments began 4–5 days after surgery.

### Recordings

Fetal MAP, ECG and EEG were recorded continuously. Fetal MAP was recorded using Novatrans II pressure transducers (MX860, Carslbad, USA) and corrected for maternal movement by subtraction of amniotic fluid pressure. The blood pressure signal was collected at 64 Hz and low-pass filtered at 30 Hz. The analogue fetal EEG signal was low-pass filtered with the cut-off frequency set with the −3 dB point at 30 Hz, and digitized at a sampling rate of 512 Hz. The intensity (power) and frequency were derived from the intensity spectrum signal between 0.5 and 20 Hz. For data presentation, the total EEG power was normalized by log transformation (dB, 20 × log intensity) and data were stored to disk as one min averages (Labview, National Instruments, Austin, TX, USA). Additionally, the unaveraged EEG signal was processed through a digital finite impulse response low-pass filter with a cut-off frequency of 30 Hz, and stored at a sampling rate of 64 Hz for analysis of seizures [13].

### Experimental Protocol

Experiments were conducted at 103–104 days of gestation. Fetuses were randomly assigned to saline-sham asphyxia (sham control, n = 8), asphyxia-saline (n = 10) or asphyxia-dexamethasone (asphyxia-DEX, n = 10) groups. We have previously reported sham control data [13], [17]. Fetal asphyxia was induced by complete umbilical cord occlusion for 25 minutes. Occlusions were started between 9:00 and 9:30 am. Fifteen minutes after the end of occlusion, ewes received a 3 ml i.m. injection of either dexamethasone (12 mg dexamethasone sodium phosphate, David Bull Laboratories, Mulgrave, Australia) or the equivalent volume of saline. The maternal weight was 60.5±1.0 kg. Recordings were started from 24 hours before asphyxia and continued for seven days after release of occlusion. Fetal arterial blood samples (0.3 ml) were taken 15 min prior to occlusion, at 5 and 17 min during occlusion, and at 10 min, 1, 2, 4 and 6 h post-occlusion, then daily thereafter between 8:30 and 9:30 am. Blood samples were analyzed for pH and blood gases (Ciba-Corning Diagnostics 845 blood gas analyzer and co-oximeter, MA, USA) and glucose and lactate levels (YSI model 2300, Yellow Springs, OH, USA).

Seven days after occlusion, ewes and fetuses were killed by an overdose of sodium pentobarbital i.v. to the ewe (9 g Pentobarb 300, Chemstock International, Christchurch, NZ), and fetal brains were perfusion fixed by gravity feed, from 1.3 m above the fetus, with 10% neutral buffered formalin and left for fixation in 10% formalin for one week, cut into 5 mm thick slices and processed for paraffin embedding using standard techniques for histological evaluation.

### Histological procedures

**Figure 1:**
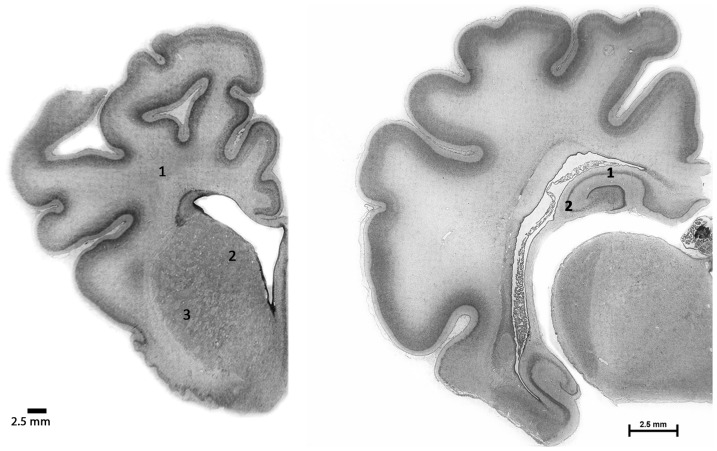
Photomicrographs of coronal sections of a preterm fetal sheep brain showing the fields used for analysis. Left panel: periventricular white matter (1) and Caudate (2) and putamen (3). Right panel: CA1-2 (1) and CA3 (2) regions of the hippocampus. Scale bars = 2.5 mm.

Histological analysis was undertaken using 8 µm thick coronal sections mounted on chrome alum-gelatin pre-coated slides, at the level of caudo-putamen and hippocampus (Figure 1). Oven dried (60 °C) slides were de-paraffinized in xylene and rehydrated in a series of ethanol solutions of decreasing concentrations, and then washed in 0.1 m PBS. Sections were stained with acid-fuchsin/thionin to assess gross morphological changes. On separate sections for immunohistochemical analysis, antigen retrieval was performed using citrate buffer (pH 6.0) using a pressure cooking method (2100 retriever, Prestige Medical Ltd, Blackburn, UK). After repeated washings in PBS the sections were treated with 1% H_2_O_2_ in methanol for 30 min in dark to quench endogenous peroxidase activity and again washed in PBS. 5% goat/horse serum in PBS was used for blocking. Antibodies were diluted with 2.5% goat/horse serum in PBS. Sections were incubated with following primary antibodies overnight at 4°C; Iba-1 (abcam; 1∶200 dilution) for activated microglia, NeuN (Millipore, 1∶200) for neuronal nuclei, Olig-2 (Millipore, 1∶200.) a marker for oligodendrocytes at all stages of the lineage [18], CNPase (abcam,1∶200), a marker for immature and mature oligodendrocytes and Ki-67 (Dako, 1.200) for proliferating cells. Washed sections were then incubated overnight with anti-goat secondary antibody for Iba-1, anti-mouse secondary antibody for NeuN and anti-rabbit secondary antibody for Olig-2 (Vector Laboratories, all 1∶200). After repeated washing with PBS, sections were then incubated with ExtrAvidin ® (Sigma, 1∶200) for 3 h at room temperature and finally treated with SIGMAFASTTM 3,3′ diamino benzidine (DAB) for brown colour development. Ki-67 labelled slides were counterstained with weak thionine solution. All slides were dehydrated in increasing alcohol concentrations and xylene, and finally mounted with DPX.

### Qualitative and quantitative analysis of brain injury

Gross morphological changes to the brain and neuronal death were assessed from acid fuchsin-thionin stained slides by light microscopy. Dead cells were identified by the characteristic acidophilic cytoplasm and pyknotic nuclei. Activated microglia and Olig-2 positive cells were estimated in periventricular white matter (PVWM, Figure 1), intragyral white matter of the cingulate gyrus (IGWM) and deep white matter (DWM, the internal capsule within the caudoputamen). Numbers of NeuN positive cells were estimated in caudate and putamen of striatum and CA1-2 & CA3 areas of hippocampus as previously described [19].

Sampling was performed using stereological principles by first tracing around each region of interest at 2× magnification, and then randomly translating a grid onto the sections and applying a fractionator probe consisting of a counting frame for object inclusion/exclusion at 40× magnification. The grid and counting frame size for the CA1/2 region were 100×100 µm and for CA3 were 50×50 µm. The grid and counting frame sizes used for the caudate nucleus and putamen were 500×500 µm and 50×50 µm, respectively. Cells touching the bottom and right-hand boundaries were included, whereas those touching the top and left were excluded. Cell counts for each region were converted to density (cells/mm^2^) by the following formula: (estimated total counts by fractionator/contour area (µm^2^))×10^6^. For each animal, average scores across both hemispheres from two sections were calculated for each region. Counts were made by an assessor blinded to treatment group.

### Data Analysis

**Figure 2:**
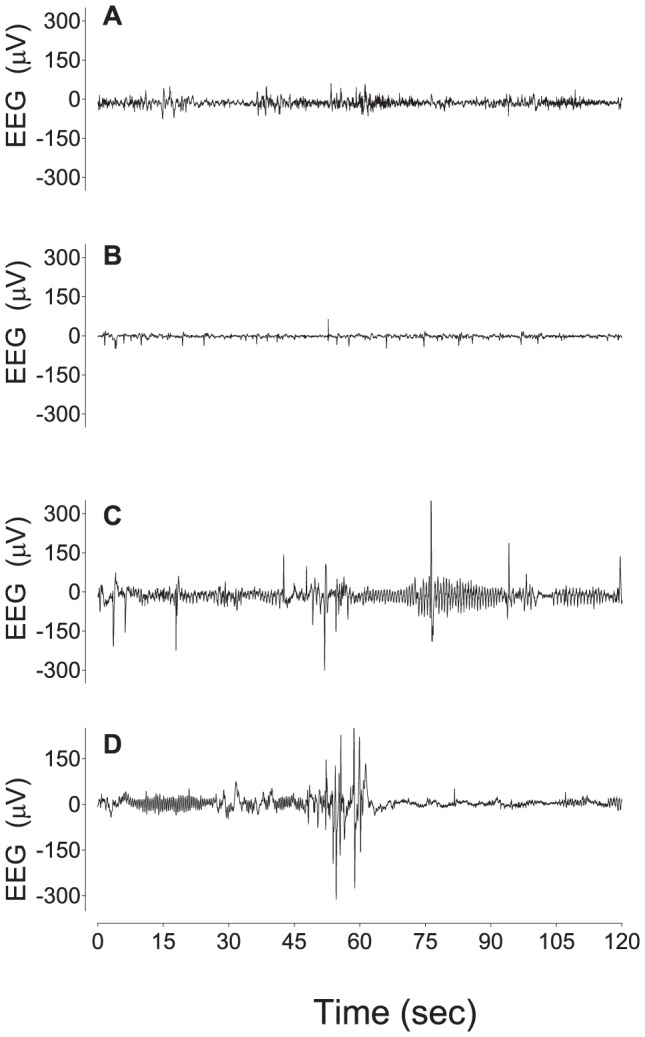
Examples of EEG patterns in preterm fetal sheep. Panel A shows an example of normal EEG activity prior to asphyxia, showing the normal mixed EEG amplitude and frequency. Panel B shows an example from the asphyxia-saline group, 4 hours after asphyxia, showing significant suppression of EEG amplitude and the presence of repetitive sharp and fast wave transients [39]. Panel C shows an example from the asphyxia-DEX group, 4 hours after asphyxia, showing the presence of interictal high amplitude slow-waves and fast waves. Panel D shows an example of a seizure-like event in the asphyxia-DEX group, 3 hours after asphyxia, characterised as slow-wave activity ending in a high amplitude burst. All figures are continuous raw data from individual animals.

Offline analysis of the physiological data was carried out using customized Labview programs (National Instruments). EEG power is expressed as change from baseline. Continuous EEG traces were analyzed for the presence of stereotypic evolving seizures defined as rhythmic, repetitive waveforms with a stereotypic evolving pattern and seizure-like activity [20]. Seizure-like events were classified as bursts of large amplitude events which had no evolving pattern or large amplitude bursts preceding or following rhythmic rolling waveforms (see Figure 2). Repetitive rhythmic slow-wave activity was quantified as% time/hour. These waveforms were defined by a period of 200–350 ms from trough to peak, with waveforms forming consistent events lasting >20 sec. These waveforms were often seen in conjunction with sharp and fast wave epileptiform transients, characterised as individual or multiple waveforms with a duration of >70 ms and <300 ms [21].

### Statistics

For analysis of changes during recovery from asphyxia, data were calculated as hour averages from the end of occlusion until the end of the experiment (SPSS v15, SPSS Inc., Chicago, Illinois, USA). The baseline was taken as the mean of the 24 h before occlusion. Treatment effects were evaluated by analysis of variance with time as a repeated measure (ANOVA, SPSS v12, SPSS Inc., Chicago, Il., USA) followed by Fisher's protected least-significant difference (LSD) post-hoc test when a significant overall effect was found. Statistical significance was accepted when P<0.05. Data are mean ± S.E.M.

## Results

### Fetal biochemistry

Umbilical cord occlusion was associated with profound hypoxemia, hypercarbia and metabolic acidosis (Table 1), which resolved progressively after release of occlusion. DEX was associated with higher fetal PaO_2_ at 1 and 2 days after occlusion, greater fetal plasma glucose from 4 to 48 h and higher plasma lactate at 4 and 6 h compared to asphyxia-saline. 4 asphyxia-DEX fetuses, but no asphyxia-saline or sham controls, went into labor within 48 h after asphyxia.

**Table 1:** Fetal blood composition data.

	Group	Baseline	5 min	17 min	+10 min	+1 h	+2 h	+4 h	+6 h	+1 day	+3 days	+5 days	+7 days
**pH**	C	7.37±0.0	7.36±0.0	7.36±0.0	7.37±0.0	7.36±0.0	7.38±0.0	7.40±0.0	7.40±0.0	7.36±0.0	7.38±0.0	7.39±0.0	7.38±0.0
	S	7.37±0.01	7.04±0.02#	6.86±0.02#	7.16±0.01#	7.29±0.01#	7.35±0.01	7.43±0.01	7.41±0.01	7.38±0.01	7.38±0.01	7.38±0.0	7.37±0.0
	D	7.38±0.00	7.05±0.02#	6.85±0.02#	7.14±0.01#	7.30±0.01#	7.34±0.03	7.37±0.02	7.38±0.02	7.38±0.02	7.35±0.1	7.36±0.0	7.37±0.0
**pCO2 (mmHg)**	C	48.0±1.5	45.0±1.4	46.1±1.3	46.1±1.3	48.5±1.6	49.4±1.6	51.3±1.2	50.3±1.2	48.8±1.5	46.1±1.9	46.2±1.6	47.0±0.8
	S	48.6±0.9	100.0±4.6#	138.2±3.2#	54.1±1.6#	43.1±1.3	45.0±1.5	43.7±0.9	46.8±0.7	47.5±0.6	47.5±0.9	47.9±1.4	48.7±1.9
	D	50.0±1.6	91.8±3.0#	131.3±5.0#	52.0±0.8#	41.2±1.3	41.0±1.0	43.0±0.8	44.4±2.1	42.1±1.1#*	48.5±1.3	46.4±1.4	49.2±0.7
**pO2 (mmHg)**	C	25.2±0.9	26.2±1.2	25.3±1.0	24.7±1.0	25.3±1.3	25.2±1.1	24.6±0.9	25.0±0.9	25.5±0.9	24.4±0.7	24.1±0.6	23.8±0.6
	S	24.1±1.2	7.1±0.9#	6.4±0.8#	35.4±1.0#	29.2±1.5	25.7±1.4	27.2±1.6	28.1±1.5#	28.3±0.8	27.1±0.9	26.3±0.9	25.2±1.0
	D	24.0±0.8	9.2±1.3#	10.0±2.1#	34.9±1.5#	32.0±2.2#	29.1±2.2#	28.5±1.0#	28.9±1.1#	31.9±0.4#*	30.7±0.8#	28.1±1.2#	29.2±1.2#
**Lactate (mmol/L)**	C	0.8±0.1	0.8±0.1	0.9±0.1	0.8±0.1	0.8±0.1	0.7±0.1	0.7±0.1	0.7±0.1	0.8±0.1	0.9±0.1	0.9±0.0	0.9±0.1
	S	0.9±0.2	4.2±0.2#	7.0±0.4#	6.4±0.3#	4.6±0.2#	3.3±0.3#	2.0±0.4#	2.2±0.4#	1.1±0.2	0.9±0.1	0.9±0.1	0.9±0.1
	D	0.8±0.1	4.1±0.3#	6.5±0.4#	6.4±0.4#	4.8±0.4#	4.1±0.7#	4.4±0.7#*	5.5±0.9#*	1.3±0.1#	0.6±0.3	0.7±0.1	0.7±0.1
**Glucose (mmol/L)**	C	1.0±0.1	0.9±0.1	1.0±0.1	1.0±0.1	1.0±0.1	1.0±0.1	1.1±0.1	0.9±0.0	1.0±0.0	0.9±0.0	0.9±0.0	0.9±0.0
	S	1.0±0.1	0.3±0.1#	0.6±0.1#	1.6±0.1#	1.3±0.1	1.3±0.1	1.3±0.2	1.4±0.2	1.1±0.1	1.0±0.1	1.1±0.0	1.0±0.1
	D	1.1±0.1	0.3±0.1#	0.6±0.1#	1.6±0.1#*	1.5±0.1	1.6±0.1#	2.5±0.1#*	3.5±0.2#*	2.1±0.2#*	1.3±0.1#	1.2±0.0	1.3±0.1#*

Data from sham controls (C), asphyxia-saline (S) and asphyxia-DEX (D) groups before occlusion (baseline), at 5 and 17 minutes during occlusion, at 10 minutes, 1, 2, 4 and 6 hours after occlusion, and daily thereafter. Days 2, 4 and 6 omitted for brevity. # P<0.05 asphyxia group compared to sham group, * P<0.05 asphyxia-saline compared to asphyxia-DEX.

### Fetal heart rate, blood pressure

**Figure 3:**
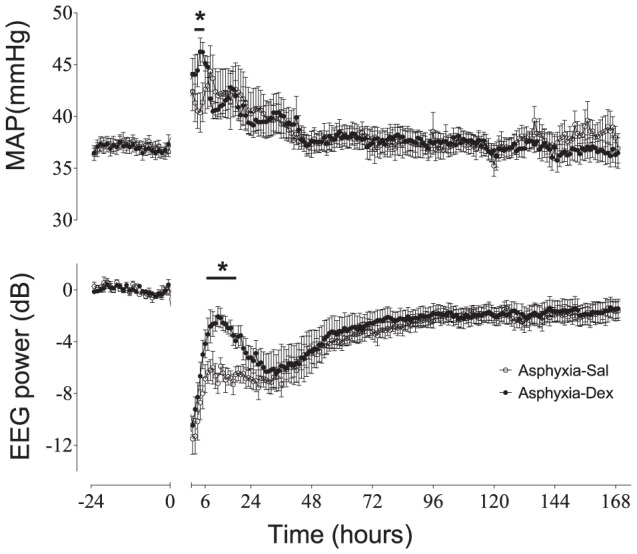
Time course of changes in mean arterial blood pressure (MAP, mmHg), and EEG power (dB) from 24 hours before, until 7 days after occlusion (occlusion data not shown) in the asphyxia-saline (open circles) and asphyxia-DEX (closed circles) groups. Data are one hour means ± SEM. *P<0.05 asphyxia-DEX vs. asphyxia-saline, # P<0.05, both occlusion groups vs. baseline.

Umbilical cord occlusion was associated with profound bradycardia and hypotension, that resolved rapidly after restoration of umbilical blood flow, with transient tachycardia in both asphyxia groups at 3–5 h (not shown, P<0.05). MAP was significantly elevated in both asphyxia groups for the first 48 h (Figure 3, P<0.05) and was transiently higher in the asphyxia-DEX group between 4–6 h (P<0.05). Thereafter there were no differences between groups.

### EEG power and seizures

After occlusion, EEG power was initially suppressed in both groups (Figure 3). EEG amplitude then increased, peaking at 9 h in the asphyxia-saline group, and 11 h in the asphyxia-DEX group. EEG power was significantly higher in the asphyxia-DEX group from 5–20 h (P<0.005). Thereafter EEG power in both groups rose progressively, but remained suppressed compared to baseline at day 7. Stereotypic evolving seizures were seen in both groups, and started earlier (7.5±0.6 vs. 14.9±4.0 h, P<0.005) and continued for longer (21.1±3.9 vs.17.7±6.4 h, P<0.05) in the asphyxia-saline group compared to the asphyxia-DEX group, but with no difference in the amplitude (171±24 vs.169±26 µV), or duration (81±7 vs. 80±5 sec/seizure) of stereotypic seizures.

**Figure 4:**
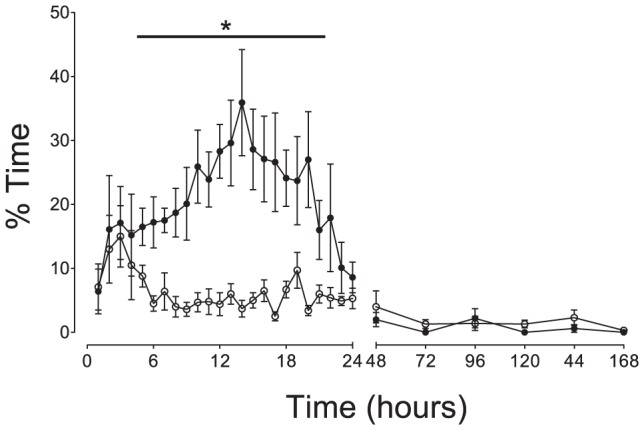
Time course of changes in% time/h of prolonged slow wave activity after asphyxia-saline (open circles) and asphyxia-DEX (closed circles). Data are one hour means ± SEM up to 24 h then 24 h averages until the end of experiment. *P<0.01 asphyxia-DEX vs. asphyxia-saline.

Analysis of the continuous EEG recordings showed that the relative increase in EEG power noted above corresponded with an increase in rhythmic slow-wave and high amplitude sharp wave transient activity (Figure 2). This activity was seen in a much greater proportion of the EEG recordings in asphyxia-DEX fetuses than asphyxia-saline fetuses during the first 24 hours after occlusion (Figure 4); post-hoc tests suggested that the groups were significantly different from 5–21 h (P<0.01). There were no differences between groups after 24 hours.

### Brain histology and immunohistochemistry

**Figure 5:**
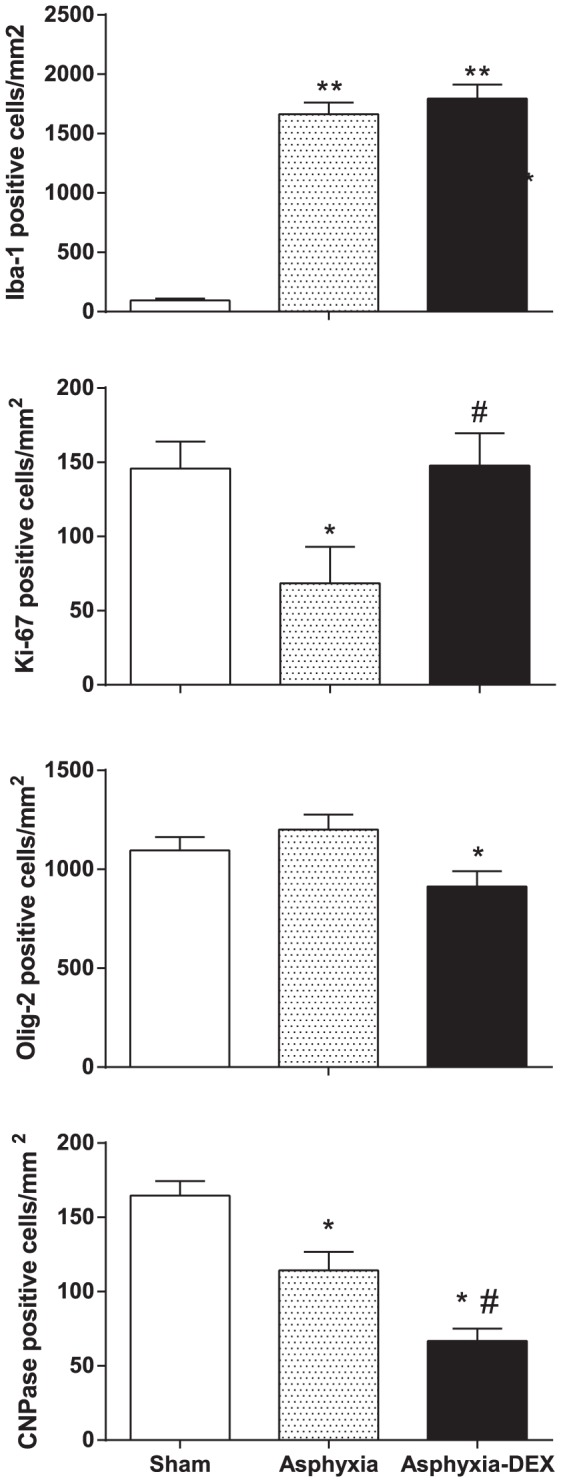
Cell counts seven days after occlusion in sham-control (white bars), asphyxia-saline (asphyxia, grey bars) and asphyxia-DEX groups (black bars), showing numbers of microglia (Iba-1), proliferating cells (Ki-67), total oligodendrocytes (Olig-2) and immature to mature oligodendrocytes (CNPase positive) in the periventricular white matter. Data are mean ± SEM. * P<0.05, ** P<0.01 compared to sham controls, # P<0.05 compared to asphyxia-saline.

No white or grey matter injury was seen in sham controls. Asphyxia was associated with marked induction of Iba-1 positive microglia in all white matter regions, which was not affected by DEX (Figure 5). In contrast, there was a marked reduction of Ki-67 labeled proliferation after asphyxia compared to sham controls. Asphyxia-DEX was associated with a significant increase in proliferation compared with asphyxia-saline, to sham control values. There was no significant change in numbers of Olig-2 positive oligodendrocytes in the PVWM in the asphyxia-saline group, in contrast with a significant reduction after asphyxia-DEX compared to both sham controls and asphyxia-saline.

There was a marked reduction in numbers of CNPase positive cells (a marker of immature and mature myelinating oligodendrocytes) in PVWM after asphyxia, with a further significant reduction in the asphyxia-DEX group. The percentage of CNPase positive oligodendrocytes in the periventricular white matter was markedly reduced after asphyxia (asphyxia-saline 9±2% vs sham controls 16±2%, P = 0.005), with no further change in the asphyxia-DEX group (9±2%).

**Figure 6:**
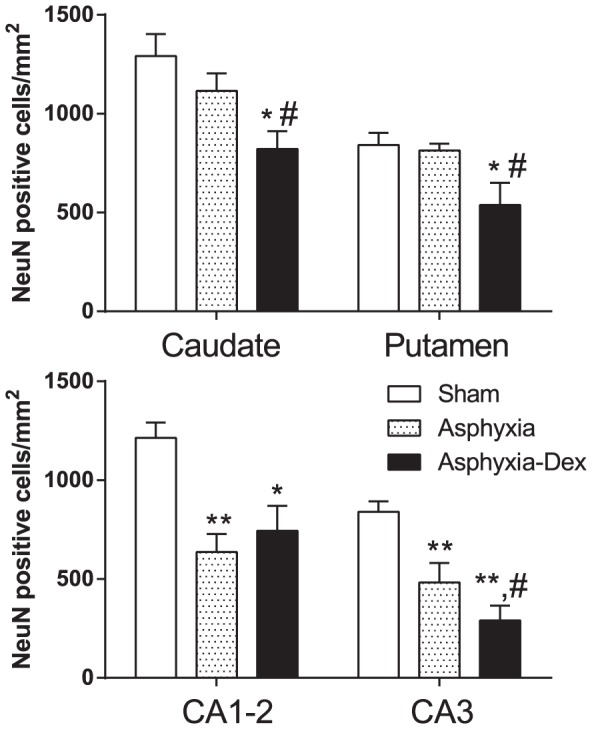
Numbers of surviving neurons (NeuN) per mm^2^ in the Caudate nucleus and the Putamen (Top) and the Cornu Ammonis regions (CA1-2 and CA3) of the hippocampus. * P<0.05, ** P<0.01 compared to sham controls, # P<0.05 compared to asphyxia-saline.

**Figure 7:**
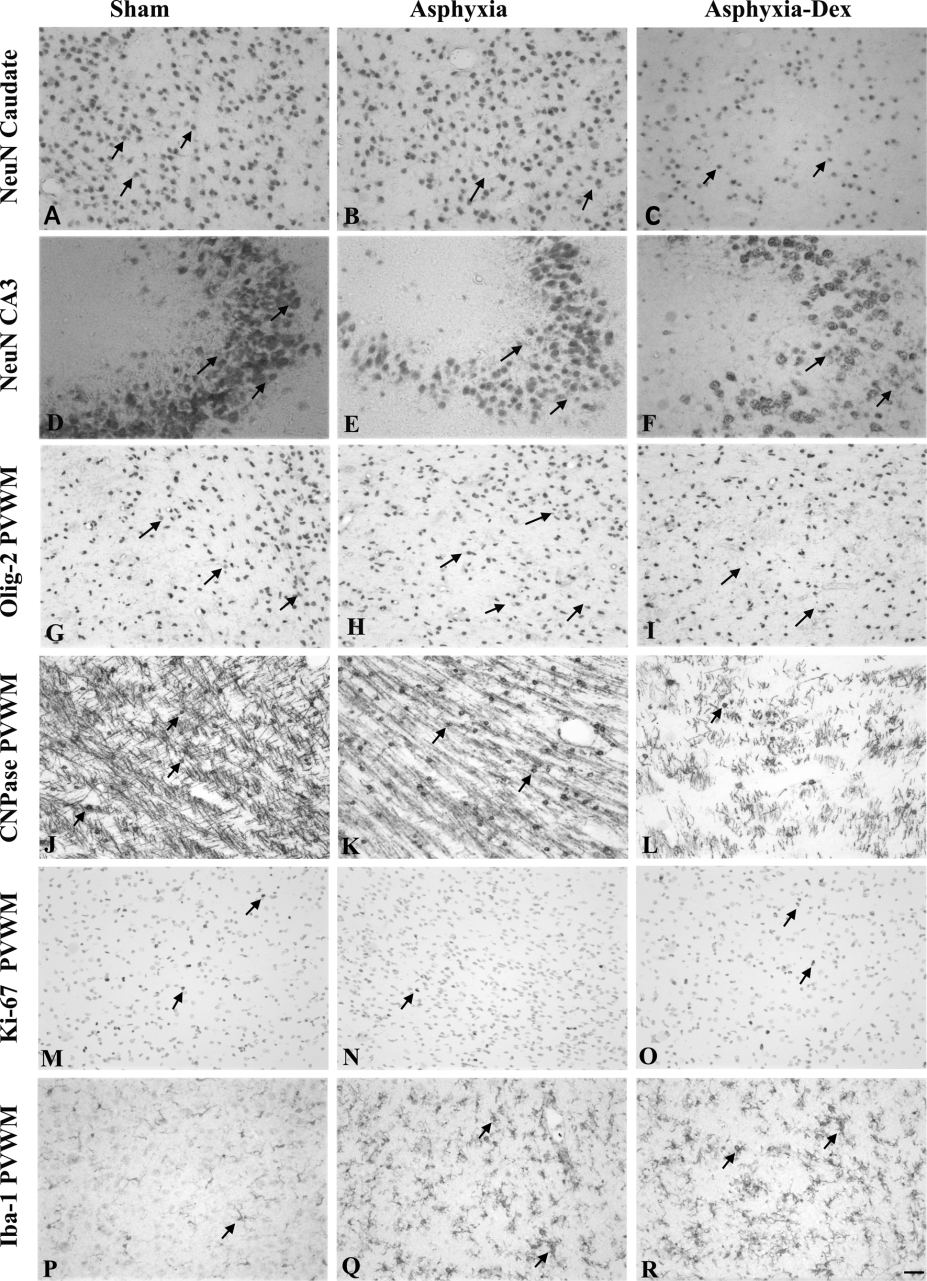
Photomicrographs showing examples of immunohistochemically stained neurons (NeuN) in the Caudate Nucleus (A-C), and in the CA3 region of the hippocampus (D-F). In Plates G to R show photomicrographs from periventricular white matter (PVWM), including all oligodendrocytes (Olig-2, G-I), immature to mature oligodendrocytes (CNPase, J-L), proliferating cells (Ki-67, M-O) and microglia (Iba-1, P-R) from sham-control, asphyxia-saline and asphyxia-DEX fetuses. Arrows show examples of labelled cells. Note the reduction in number of neurons and oligodendrocytes after asphyxia and further loss with DEX treatment, and marked induction of microglia in the PVWM. Scale bar is 40 µm.

Asphyxia-saline was associated with marked neuronal loss in the hippocampal regions but not the caudoputamen (Figures 6 & 7). Asphyxia-DEX was associated with significantly greater neuronal loss overall compared with both sham controls and asphyxia-saline. Post-hoc analysis showed significantly greater neuronal loss in the putamen and the CA3 region of the hippocampus after asphyxia-DEX compared with asphyxia-saline (P<0.05).

## Discussion

This study demonstrates, for the first time, that dexamethasone treatment of pregnant ewes shortly after *in utero* asphyxia was associated with greater subcortical neuronal loss and white matter injury as shown by loss of oligodendrocytes in the periventricular white matter tracts in preterm fetal sheep. During recovery from asphyxia, from 6 to 24 h after occlusion, maternal dexamethasone was associated with a marked transient increase in background fetal EEG power, reflecting mainly abnormal slow-wave activity, but delayed onset of seizures. Further, fetal plasma glucose levels were significantly greater during both the latent and secondary phases after maternal DEX treatment, with a transient increase in fetal lactate.

Maternal glucocorticoid therapy is now standard care for threatened premature delivery, including cases where the fetus is at risk of hypoxia. There is surprisingly little information on the potential impact of antenatal steroids on fetal or newborn brain injury [11]. In postnatal day 7 rats, dexamethasone after HI did not affect infarct size [22], [23], although there was increased neuronal apoptosis raising the possibility of greater long-term injury. Similarly, studies in adult animals suggest little effect of dexamethasone given after cerebral ischemia [11], although in one study prolonged post-insult treatment reduced injury in the caudate nucleus, but no other regions [24]. Strikingly, there are no translational studies in fetal animals of steroids given after HI.

Periventricular white matter damage is highly characteristic of preterm brain injury. In this study we found no net loss of total (Olig-2 positive) oligodendrocytes in the periventricular white matter after asphyxia and only a minor reduction in numbers in fetuses exposed to maternal dexamethasone. In contrast, there was a marked reduction in both numbers and the proportion of immature/mature myelinating oligodendrocytes in the periventricular white matter after asphyxia-saline. There was a further reduction in numbers of myelinating oligodendrocytes after asphyxia-DEX, although interestingly, the reduction in their percentage of total oligodendrocytes in the PVWM was similar to asphyxia-saline. We have previously shown marked loss of pre-oligodendrocytes 3 days after asphyxia in the same paradigm as the present study [25]. This combination of findings is consistent with the seminal report of extensive restorative proliferation after hypoxic-ischemic death of pre-oligodendrocytes in the developing brain, followed by chronically impaired maturation [9]. At day 7 after injury we cannot determine whether DEX exacerbated acute loss of white matter cells, however, it is highly likely that it impaired restorative proliferation, consistent with the finding of suppressed fetal neural proliferation after maternal glucocorticoids in rats [26]. The increased Ki-67-labelled proliferation in white matter by day 7 in this study is consistent with the delayed compensatory rebound increase in proliferation seen up to three weeks after maternal glucocorticoid treatment in the rat [26]. Further long-term studies are needed to determine whether this impaired maturation will resolve with time.

The mechanisms by which maternal glucocorticoids increased fetal neural injury in the present study are likely multifactorial. Increased injury was not related to post-asphyxial hypotension. Although dexamethasone is a potent anti-inflammatory agent, there was no effect on microglial induction by day 7 [11]. As previously reviewed [27], even after surprisingly severe insults there can be transient normalization of oxidative metabolism in a critical ‘latent’ phase when cell survival can be modulated, followed by a secondary phase of progressive cell death hours to days after reperfusion. Loss of neural suppression in the latent phase can increase neural injury [14], [15]. In this study, there was no change in EEG activity in the first 6 hours suggesting that neuronal recovery in the latent phase was not compromised. However, in the secondary phase maternal dexamethasone treatment was associated with a marked increase in background fetal EEG power compared to continued suppression of background EEG power in the asphyxia-saline group. This was not due to increased seizures but to greater interictal activity. Since, glucocorticoids can enhance glutamate release [28], this suggests the hypothesis that dexamethasone increased delayed excitotoxicity in the present study. Conversely, the onset of stereotypic seizures was delayed. Clinically and experimentally, delayed onset of seizures is associated with more severe injury [29]–[31]. Thus, this finding is consistent with the greater neural injury in the present study [11], [32], [33].

Finally, it is possible that the increase in plasma glucose levels in the latent phase and later may contribute to greater injury. As recently reviewed, the effect of blood sugar on ischemic injury is complex [11]. Clinically it is highly controversial whether controlling glucose affects outcomes after ischemia [34]; however, hyperglycemia in preterm infants is associated with increased morbidity and mortality [35]. The fetus is an obligatory glucose user and hyperglycemia increases glucose utilization in the fetus [36], potentially increasing metabolism. In newborn piglets, although hyperglycemia during HI exacerbated brain injury, hyperglycemia after HI similar to that seen in the present study did not affect the severity of neural injury [37].

The present study examined the effects of dexamethasone, one of the two major synthetic glucocorticoids used for antenatal treatment. As previously reviewed, it is unclear whether there is any material difference in neonatal outcomes between dexamethasone and betamethasone, despite these compounds differing only in the orientation of one methyl group [11], [32]. Both are widely used [32]. Potentially, glucocorticoids may have sex specific effects in some situations [26], [38]. The current study was not powered to evaluate this possibility, although the study groups had similar numbers of male and female fetuses (Table 2). Further, there is potential for much longer term effects of glucocorticoids than addressed in this study [33]. Given this controversy, it will be important to directly compare the effects of these glucocorticoids in future studies and to assess their long-term impact on brain development and function and whether they may have sex specific effects.

**Table 2:** Postmortem data.

	Body Weight (g)	Brain weight (g)	Sex (m:f)
**C**	1950±148	30.5±2.7	4∶4
**S**	2065±128	28.6±1.4	5∶5
**D**	1933±105	27.3±0.7	6∶4

Sham controls (C), asphyxia-saline (S) and asphyxia-DEX (D) groups.

In conclusion, the present study shows that a clinical dose of maternal dexamethasone given shortly after asphyxia was associated with increased periventricular white matter damage and neuronal loss in the putamen and the CA3 region of the hippocampus, and increased interictal fetal EEG activity during recovery. These findings suggest that maternal glucocorticoid therapy can adversely affect recovery of the immature brain after hypoxia-ischemia.
